# Impact of Lactic Acid Bacteria Fermentation Based on Biotransformation of Phenolic Compounds and Antioxidant Capacity of Mushrooms

**DOI:** 10.3390/foods13111616

**Published:** 2024-05-23

**Authors:** Eda Nur Ayar-Sümer, Yannick Verheust, Beraat Özçelik, Katleen Raes

**Affiliations:** 1 Research Unit VEG-i-TEC, Department of Food Technology, Safety and Health, Faculty of Bioscience Engineering, Ghent University, St-Martem Latemlaan 2B, 8500 Kortrijk, Belgium; ayare15@itu.edu.tr (E.N.A.-S.); yannick.verheust@ugent.be (Y.V.); 2 Department of Food Engineering, Faculty of Chemical and Metallurgical Engineering, Istanbul Technical University, Maslak, TR-34469 Istanbul, Turkey; ozcelik@itu.edu.tr

**Keywords:** fermented mushrooms, lactic fermentation, bound phenolics, free phenolics, bioactive compounds, antioxidant activities, UPLC-Q-TOF-MS/MS

## Abstract

Mushrooms contain phenolic compounds that possess health-promoting properties, including antioxidant effects. However, the low solubility and form of phenolic compounds affect their bioactivity and bioaccessibility. To overcome this limitation, our study investigates the fermentation of mushrooms to increase their free phenolic content and enhance their bioactivity. Our research focused on the impact of fermentation on both free and bound phenolic fractions (FPs and BPs, respectively) in *Lentinula edodes* and *Lactarius deliciosus*, which were successively fermented with *Lactiplantibacillus plantarum LMG 17673* for 72 h. We examined the total phenolic content (TPC), phenolic profile, and antioxidant activity of both FPs and BPs. Our results showed that the TPC of BPs was higher than that of FPs in both mushrooms, with strong antioxidant capabilities. Fermentation significantly increased the TPC of FPs in both mushrooms, particularly after 24 h of fermentation. The TPC of BPs in mushrooms decreased during fermentation, indicating their release from the matrix. Additionally, we identified 30 bioactive compounds using UPLC-Q-TOF-MS/MS. Our study demonstrates for the first time that lactic acid bacteria fermentation of mushrooms with high phenolic content leads to the liberation of bound phenolics, enhancing their bioactivity and bioaccessibility.

## 1. Introduction

Consumption of edible mushrooms (cultivated and wild varieties) has been a global practice for centuries. *Lentinula edodes*, commonly referred to as shiitake mushrooms, is the second most cultivated edible mushroom worldwide [1]. In Europe, the commercialisation of 268 distinct edible wild mushroom species has been officially sanctioned, and among these, *Lactarius deliciosus* is one of the top five best-selling varieties. Recognised as saffron milkcap, *La. deliciosus* is a popular and extensively consumed wild edible mushroom [2,3].

The global mushroom cultivation market size is expected to reach approximately 24 million tons by 2028, which is 57% higher than that of 2021 [4]. However, this growth poses environmental challenges for the market because of the short shelf life of the mushrooms. In addition, a significant amount of by-products are produced during mushroom production, leading to high environmental impact and management costs for the industry. These by-products consist of caps, stipes, mushrooms that do not meet commercial standards for size, shape, or calibration, and spent mushroom substrates. Approximately 5 kg of spent mushroom substrate by-products are generated per kilogram of fresh mushroom produced [5], whereas up to 20% of fresh mushroom production volume is attributed to misshaped mushroom by-products [6]. The misshapen mushrooms have basically the same chemical composition as that of the normal mushroom [7]. Therefore, applying food processing to mushrooms may not only serve as an effective means of valorisation to by-product mushrooms but also contribute to extending the shelf life of all mushrooms.

Consumers are becoming interested in the potential health benefits of adding mushrooms to their diet. Mushrooms are recognised for their nutritional value as plant-based protein sources containing essential vitamins, minerals, fibre, and antioxidants. Additionally, mushrooms contain bioactive compounds with anti-inflammatory, antioxidant, antitumour, antiviral, and antimicrobial properties that actively promote health and reduce the risk of diseases in the human body [8]. These positive effects on health are due to the presence of bioactive compounds, such as phenolic compounds, terpenoids, steroids, lectins, nucleotides, glycoproteins, and polysaccharides [9], and much attention has been given to phenolic compounds as secondary metabolites in mushrooms [10]. Phenolic compounds are usually present in food matrices in both free and bound forms. As the bound phenolic compounds are linked to the cell wall matrices with covalent bonds, they cannot be absorbed in the small intestine, resulting in lower bioavailability compared to the free phenolic compounds. To increase the bioavailability of phenolic compounds, it is necessary to use additional processing techniques, such that bound phenolic compounds can be released and become available for absorption, similar to free phenolic compounds. This would also contribute to enhancing the health benefits of mushrooms [11].

Fermentation is a traditional non-thermal food processing method that involves the metabolic activities of microorganisms such as bacteria and fungi. Lactic acid bacteria (LAB) are gram-positive bacteria known for their ability to convert sugars to lactic acid during food fermentation. In addition to increasing the shelf life and improving the sensory qualities of food, LAB degrades macronutrients such as carbohydrates and proteins, resulting in alterations in the nutritional composition of the food product [12]. This process results in food biotransformation by converting macronutrients, releasing antioxidative peptides, and altering phenolic compounds [13]. In particular, *Lactiplantibacillus plantarum LMG 17673*, a versatile and widely utilised LAB species, is particularly effective in fermenting fruits, vegetables, and dairy products, with beneficial effects on human health [14].

Many studies have investigated the antioxidant activity properties of cultivated and wild edible mushrooms. However, to our knowledge, no research has been conducted on the impact of LAB fermentation, as a possible processing technique for mushrooms, on the free and bound phenolic compounds in mushroom bodies. Since phenolic compounds are diverse and have complex structures, advanced analytical techniques are needed for which the ultra-performance liquid chromatography–electrospray ionisation quadrupole time-of-flight mass spectrometry (UPLC-ESI-QTOF-MS/MS) method is commonly used to tentatively identify extracts. In addition, this study is the first to use lactic acid bacteria and fermentation to enhance the antioxidant activity of mushrooms by liberating the bound phenolic compounds.

## 2. Materials and Methods

### 2.1. Media and Chemicals

De Man-Rogosa-Sharpe (MRS) and plate count agar were purchased from Oxoid LTD (Basingstoke, Hampshire, England). Folin-Ciocalteu reagent, DPPH (2,2-diphenyl-1-picrylhydrazyl), ABTS (2,2′-azinobis-(3-ethylbenzothiazoline-6-sulphonic acid)), trolox (6-hydroxyl-2,5,7,8-tetramethyl chroman-2-carboxylic acid), gallic acid, vanillic acid, salicylic acid, L-rhamnose monohydrate, DL-arabinose, D(+)-xylose, D(+)-mannose, D(−)-ribose, D(+)-glucose, D(+)-maltose monohydrate, D(−)-mannitol, trehalose, galacturonic acid, and glucuronic acid were purchased from Sigma–Aldrich Fine Chemicals (St. Louis, MO, USA). D(−)-Fructose was purchased from Acros Organics (Geel, Belgium). HPLC-grade water and methanol were purchased from VWR Chemicals (VWR International S.A.S., Briare, France). Aluminium chloride, sodium nitrite, sodium hydroxide, sodium carbonate, hydrochloric acid, and methanol were purchased from Chem-Lab (ChemLab NV, Zedelgem, Belgium).

### 2.2. Sample Preparation and Lactic Acid Fermentation

In this study, although misshapen mushroom by-products were the preferred source for fermentation, our experimental outcomes remained unaffected by the use of either mushrooms or their misshapen by-products, as their nutritional compositions were identical. Due to practical considerations, fermentation was performed directly with mushrooms, which was more feasible than obtaining them from the mushroom by-product. Therefore, *L. edodes* and *La. deliciosus* were purchased from a local market in Turkey, known for their high standards and reliability, to guarantee the correct identification of the mushrooms. Furthermore, the mushrooms bought were controlled for their identification based on the mushroom identification guides as described by Davis et al. (2013) and Stamets (2011) and thoroughly examined for their key morphological features (cap shape, colour, size, and spore print) [15,16]. The mushrooms were cleaned before being cut into small pieces measuring 1 × 1 cm, frozen at −20 °C, and lyophilised (Christ Alphna 1-2 LDplus, Osterode am Harz, Germany). These lyophilised samples were then finely ground into a mushroom powder using a grinder (IKA A11, Staufen, Germany). The mushroom powder was mixed with physiological water to obtain a 2% (*w*/*v*) mushroom solution and sterilised.

The lactobacillus strain of *Lactiplantibacillus plantarum LMG 17673* was purchased from the BCCM/LMG Bacteria Collection, an integral component of the Belgian Co-ordinated Collections of Microorganisms situated within the Laboratory for Microbiology of the Faculty of Sciences of Ghent University, Belgium. The inoculum of the strain was prepared according to the method described by He et al. (2021) with slight modifications. The lactobacilli culture was aseptically activated by transferring 100 μL of glycerol stock culture into 10 mL of sterile MRS broth and was then incubated at 30 °C for 24 h [17]. *Lp. plantarum LMG 17673* was propagated in MRS broth at 30 °C for 24 h with shaking at 110 rpm before being used as a working culture to inoculate the mushroom solutions. Flasks containing 25 mL of sterilised mushroom solution were inoculated with 1% (*v*/*v*) working culture and then placed on a rotary shaker (110 rpm) at 30 °C. During fermentation, samples were collected at different time points (0, 24, 48, and 72 h), freeze-dried, and the powders were stored at −20 °C until further analysis.

### 2.3. Microbial Analysis

Microbial composition during fermentation was evaluated by measuring the pH and counting the number of LAB. The standard plate count method analysed LAB counts in mushroom samples at specific time points (0, 24, 48, and 72 h). Serial dilutions were prepared using physiological water. Using the spread plate method, 100 μL diluted aliquots were plated onto MRS agar plates and then cultured at 30 °C for 48 h. Finally, the microbial population was expressed as logarithmic colony-forming units per mL (log CFU/mL).

### 2.4. Fermentation Metabolites

The fermentation metabolites of mushrooms were quantified at specific time points (0, 24, 48, and 72 h) using pre-column high-performance liquid chromatography (HPLC) [18]. The metabolite analysis employed an LC system (Agilent LC 1260 Infinity II, Gent, Belgium) equipped with a column (Agilent Hi-Plex 300 × 7.7 mm, 8 μm particle, Gent, Belgium). The column was maintained at 60 °C, the diode array detector was set to 210 nm, and the refractive index detector was set to 55 °C. A fixed flow rate of 0.7 mL/min was combined with a 5 mmol/L sulfuric acid mobile phase, and 20 μL injection volumes were used. Samples were prepared by mixing 1 g of fermented mushrooms with 5 mL of 5 mmol/L sulfuric acid using an Ultraturrax (IKA-T18, Staufen, Germany) for 1 min at 10,000 rpm at 21 °C. Then, the probe was washed with 5 mL of 5 mmol/L sulfuric acid and centrifuged (Hermle Z 366 K, Wehingen, Germany) at 4000 rpm for 10 min at 21 °C. The resulting mixture was then filtered through a 0.45 μm disc filter, and the supernatant was collected and preserved at −40 °C until analysis. The quantification of free sugars and organic acids was based on calibration curves established for each compound by injecting known concentrations of external standards. Finally, the results were expressed as mg per g of dry fermented mushrooms. In this study, the total free sugar compound refers to the sum of determined glucose, ribose, trehalose, and mannitol, whereas the total organic acid refers to the sum of specified formic acid, malic acid, lactic acid, citric acid, fumaric acid, and succinic acid.

### 2.5. Extraction of Free and Bound Phenolic Fractions

The method by Gonzales et al. (2014) [19] was used to extract the free and bound phenolics. In summary, 2 g of fermented mushroom powder were blended with 15 mL of 100% methanol using Ultra-Turrax (IKA-T18D, Staufen, Germany) at 3000 rpm for 45 s. The tubes were promptly cooled in an ice bath for 15 s before being centrifuged at 13,000× *g* for 10 min at 4 °C using a centrifuge (Z 300 K, Hermle Labortechnik GmbH, Wehingen, Germany). The residue was subjected to a second extraction with 10 mL of 80% methanol, using the same procedure as before. The resulting supernatant was filtered, and the volume was adjusted to 25 mL using 80% methanol. The phenolic content of these extracts was referred to as the free phenolics (FPs). The residue was air-dried in a fume hood overnight to extract the bound phenolics. Briefly, 0.1 g of the air-dried residue was subjected to hydrolysis by adding 2 mL of 2M NaOH using ultrasound (UP 400S, Hielscher, GmbH, Chamerau, Germany) for 30 min at maximum amplitude (100%) and 60 °C. The hydrolysed samples were neutralised with 2M HCl, followed by extraction with 4 mL of methanol containing 0.1% formic acid. After vortexing and centrifugation at 10,000× *g* for 10 min at 4 °C, the residue underwent a second extraction with 4 mL of 0.1% formic acid in 100% methanol. Finally, the supernatants from both extractions were combined and adjusted to 20 mL with 80% methanol. The phenolic content of these extracts is referred to as the bound phenolics (BPs).

### 2.6. Total Phenolic Content

The total phenolic content (TPC) of the free and bound phenolic extracts was determined using the Folin–Ciocalteu method [20]. Each extract (1 mL) was mixed with 0.5 mL of 10-fold diluted Folin–Ciocalteu reagent and then neutralised with 1.5 mL of 20% sodium carbonate solution. The mixture was then incubated for 2 h at room temperature. After incubation, the blue colour that formed was measured using a 760 nm spectrophotometer (Model 4001/4, Thermo Spectronic, Waltham, MA, USA). The TPC was calculated using gallic acid calibration curves with R^2^ values > 0.99 and expressed as mg gallic acid equivalent (GAE) per gram of mushroom dry matter (dm). The limit of detection (LOD) and the limit of quantitation (LOQ) were determined to be 0.16 and 1.44 μg GAE/mL, respectively.

### 2.7. Antioxidant Properties

#### 2.7.1. Determination of DPPH Free-Radical-Scavenging Activity

To evaluate the antiradical activity against DPPH radicals, the method devised by Kumaran and Karunakaran [21] was applied. Briefly, a 0.1 mM DPPH solution (2 mL) was mixed with 100 μL of the extracts by vortexing for 10 s. Then, the mixture was incubated in the dark for 30 min at room temperature and the absorbance of the solution was measured at 517 nm using a spectrophotometer. The DPPH free-radical-scavenging capacity was calculated using the Trolox calibration curve with R^2^ > 0.99 and expressed as mg Trolox equivalent (TE)/100 g mushroom dm. LOD and LOQ were found to be 2.6 and 7.01 μg TE/mL, respectively.

#### 2.7.2. Determination of ABTS Free-Radical-Scavenging Activity

ABTS radical-scavenging capacity was assessed according to the method described by Re et al. [22]. ABTS was dissolved in distilled water to create a stock solution with a concentration of 7 mM. Then, the reaction between the ABTS stock solution and potassium persulfate (2.45 mM) was initiated to generate an ABTS radical cation, which was left in the dark at room temperature for 12–16 h before utilisation. The ABTS radical cation was diluted with 90% methanol to prepare a working solution of fresh ABTS radical cation, achieving an absorbance of 0.70 ± 0.02 at 734 nm and incubating at 30 °C. Subsequently, 2 mL of fresh ABTS radical cation solution was mixed with 20 μL of sample extract and vortexed. Afterwards, the mixture was incubated for 5 min in the dark at 25 °C and the absorbance of the solution at 734 nm was measured. The results were calculated using the Trolox calibration curve with R^2^ values > 0.99. and expressed as mg Trolox equivalent (TE)/g mushroom dm. LOD and LOQ were found to be 2.74 and 8.23 μg TE/mL, respectively.

### 2.8. Phenolic Compound Identification and Characterisation

#### 2.8.1. UPLC-Q-TOF-MS/MS-Based Metabolite Analysis

Fermentation metabolomic measurements were performed using an ultra-high-performance liquid chromatography system (UPLC Infinity 1290, Agilent Technologies, Santa Clara, CA, USA) coupled to a quadrupole time-of-flight mass spectrometer (Q-TOF 6546, Agilent Technologies) [23]. The system was equipped with an online degasser (Model 590, Alltech elite degassing system, Agilent Technologies, Santa Clara, CA, USA), a sampler (G 7129B), a quaternary pump (G 7104A), Column18 5u (4.6 × 150 mm; GRACE, Deerfield, IL, USA), Column Oven (G 7130A), and photodiode array detector (DAD) (G 7117A, Agilent Technologies). Separation was carried out using a gradient elution method with two mobile phases: ultrapure water (0.1%, *v*/*v*, formic acid; eluent A) and LC-MS-graded methanol (0.1%, *v*/*v*, formic acid; eluent B). The gradient profile was as follows: 0–6 min, isocratic 20% B; 6–12 min, isocratic 20% B; 12–13 min, 20–30% B; 13–23 min, 30–50%; 23–30 min, 50–90%; 30–35 min, isocratic 90%; 35–40 min, 90–10%; and 40–45 min, isocratic 10%. Each sample was run for a total of 45 min at a flow rate of 0.25 mL/min and an injection volume of 20 µL. The eluent was directed into a Dual Agilent Jet Stream (AJS) electrospray ionisation (ESI) source within the mass spectrometer. The parameters for the MS were set as follows: capillary voltage, 3.5 kV; nozzle voltage, 4 V; gas temperature, 300 °C; vaporiser temperature, 350 °C; gas flow rate, 8 L min^−1^; and vaporiser flow, 11 L min^−1^. A complete mass scan covering *m*/*z* 100–1700 was performed. For target analysis and fragmentation, MS/MS analysis was performed in the negative mode at different collision energies of 10, 20, and 40 V. Instrument control, data acquisition, and processing were performed using Agilent MassHunter Workstation Qualitative Analysis software (version 10.0, Agilent Technologies, Santa Clara, CA, USA).

#### 2.8.2. Non-Target Screening on UPLC-Q-TOF-MS

All samples were analysed in triplicate in the negative mode. The acquisition procedure followed the described protocol, utilising Agilent MassHunter Workstation Profinder software (version 10.0) to extract non-target compounds via the Batch Recursive Feature Extraction workflow. Initially, the software identified molecular features in the first sample, where each feature represented a group of corresponding ions, including isotopes and adducts of the same compound. These groups formed a chromatographic peak at a specific retention time (RT). These detected features, accompanied by their precise monoisotopic mass and RT, were stored in a list, serving as a reference source for re-mining sample data using the Find-by-Ion method. Subsequently, the software used a binning algorithm to scan all the features across the samples. Detected features from all samples were compared, aligning exact masses and RT, and grouping corresponding features. This process generated a comprehensive list of features and their presence in respective samples. In a subsequent step, the average exact masses and RT of the consensus feature list were more sensitively scanned in each sample to detect any missed features in the initial round, facilitating recursive feature extraction. This approach enhances the feature identification accuracy by searching for low-level yet high-quality features that may have been overlooked initially, thus reducing false negatives and accelerating the binning process.

#### 2.8.3. Non-Target Metabolomics Analysis

The acquired feature list was imported into Agilent Mass Profiler Professional (MPP, version 15.1, Agilent Technologies), which offers a complete array of advanced statistical tools and facilitates the powerful visualisation of the results. Due to the impracticality of identifying all features, statistical analyses were employed to focus on the relevant compounds. Initially, principal component analysis (PCA) was used to assess metabolic variations among replicate samples. Following this, Fold Change analysis was applied to features demonstrating abundance variations between different conditions. Paired analyses were conducted using the Benjamini–Hochberg method, and the findings were visually represented through volcano plots.

#### 2.8.4. Tentative Identification of Phenolic Compounds

Qualitative analysis of phenolic compounds in fermented mushroom fractions was carried out using UPLC-Q-TOF-MS/MS in negative ionisation mode. The Agilent MassHunter Workstation Qualitative Analysis software (version 10.0) was used to process the data and extract additional details about the features of interest through a target MS/MS approach. The acquired MS/MS spectra are compared with the METLIN Agilent MassHunter Personal Compound Database and Library (PCDL) via the find-by-formula search feature. The software tentatively identifies target compounds by matching observed spectra to reference spectra. Only compounds with good matching (score > 75/100) and mass errors within ±5 ppm of the metabolite results were tentatively identified. The software evaluates the similarity or match quality between the observed MS/MS spectra and reference spectra from the library and assigns scores to these matches. Identification of compounds not found in the library was attempted again using MS/MS data from the ChemSpider database. The MS/MS data of the available authentic standards were also compared with the experimentally measured mass of the ions for verification.

### 2.9. Statistical Analysis

Correlations between TPC and antioxidant activity were evaluated using Pearson’s correlation coefficient test. Statistical analyses were performed using the SPSS version 28.0 program (IBM SPSS Statistics, SPSS Inc., Chicago, IL, USA). To examine variations through mushroom fermentation, a one-way analysis of variance (ANOVA) was used. Subsequently, a multiple range test, Tukey’s Honestly Significant Difference (HSD) test, was used to compare the means and identify significant differences among them. A significance level of *p* < 0.05 was employed to determine the presence of statistically significant differences. The reported values are presented as the mean ± standard deviation (SD) based on three independent samples.

## 3. Results and Discussion

### 3.1. The Bacterial Growth and pH Changes during Mushroom Fermentation

**Figure 1:**
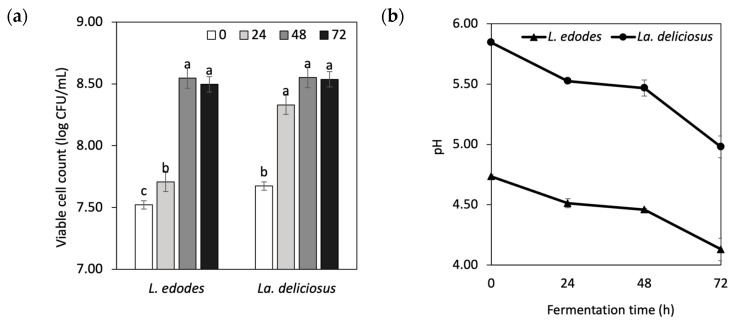
Microbial activity of *Lp. Plantarum LMG 17673*. (**a**) Viable cell count and (**b**) pH throughout the fermentation of *L. edodes* and *La. Deliciosus.* CFU: colony forming unit. Different lowercase letters within the same mushroom species indicate significant differences during fermentation (*p* < 0.05). Values are presented as mean ± SD (*n* = 3).

The changes in the viable cell count and pH values of the fermented mushrooms during the 72 h fermentation period are shown in Figure 1. As shown in Figure 1a, *Lp. plantarum LMG 17673* demonstrated robust growth in the mushroom samples, with no significant changes in viable cell count observed during the latter stages of fermentation (from 48 to 72 h) (*p* ≥ 0.05). Initially, the viable bacteria counts of *Lp. plantarum LMG 17673* in *L. edodes* and *La. deliciosus* were 7.52 ± 0.07 and 7.67 ± 0.06 log CFU/mL, respectively. After 24 h of fermentation, both mushrooms exhibited a high growth rate, which remained stable throughout the fermentation process. In the later stages (from 48 to 72 h), the viable cell counts of *Lp. plantarum LMG 17673* remained above 8.5 log CFU/mL, indicating sustained metabolic activity. These findings confirm that the selected mushrooms are suitable substrates for the fermentation of the particular species.

During fermentation, LAB strains can metabolise sugar and produce organic acids, leading to changes in the pH value of mushrooms. The pH changes of mushroom samples during fermentation with *Lp. plantarum LMG 17673* are shown in Figure 1b. The most rapid decrease in pH occurred during the initial 24 h fermentation, dropping to 4.51 and 5.53 for *L. edodes* and *La. deliciosus*, respectively. These results are comparable to the pH of 4.61 observed in pickled *Agaricus bisporus* fermented with *Lb. plantarum* [24]. A lower pH inhibits the growth of pathogenic bacteria, potentially extending shelf life and maintaining anaerobic conditions for preservation [25]. Additionally, the pH reduction during fermentation is attributed to the accumulation of organic acids, as noted in recent studies [26].

### 3.2. Changes in the Organic Acid and Sugar Levels during Mushroom Fermentation

**Figure 2:**
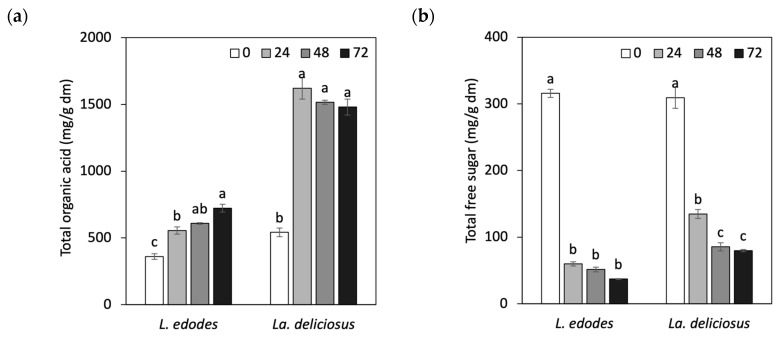
Effect of *Lp. plantarum LMG 17673* fermentation on mushrooms. (**a**) Total free sugar content and (**b**) total organic acid content. Different lowercase letters within the same mushroom species indicate significant differences during fermentation (*p* < 0.05). Values are presented as mean ± SD (*n* = 3).

The changes in organic acids and free sugars in fermented mushrooms are presented in Table S1. During mushroom fermentation, *Lp. Plantarum LMG 17673* can produce organic acids that lower the pH of the fermented mushroom matrixes. The organic acid contents of formic acid, malic acid, lactic acid, citric acid, fumaric acid, and succinic acid were assessed. During fermentation, the total organic acid content first increased, followed by a period of slight increase or stability (Figure 2a). The first 24 h of fermentation marked a crucial point for the alteration in organic acid content. Throughout the fermentation, there was an increase in lactic acid and formic acid levels and a corresponding decrease in malic acid, citric acid, fumaric acid, and succinic acid concentrations. These trends are consistent with a study by Yang et al. (2022), who fermented apple juice using LAB strains [27]. Malic acid can be transformed into lactic acid with the help of the malolactic enzyme produced by LAB, whereas the decomposition of citric acid may cause decreased citric acid content in various products. The fumaric and succinic acid contents in the fermented samples follow similar trends to those of malic and citric acids.

The main mushroom monosaccharides, disaccharides, and sugar alcohols were glucose, ribose, trehalose, and mannitol, respectively, as has been reported for other edible mushrooms [28]. During the 72 h fermentation of mushrooms, the free sugar concentration in mushrooms decreased as shown in Figure 2b, primarily due to sugar consumption by LAB [29]. Previous studies have also reported decreased free sugar content in fermented foods such as cabbage during the initial fermentation stage [29]. Glucose and trehalose were rapidly consumed during the initial 24 h, while ribose was the only sugar found in the mushrooms after 72 h, similar to that reported for button mushrooms [24]. At the beginning of the fermentation process, mannitol, a sugar alcohol, was the most abundant sugar in the fermented mushrooms. After 72 h of fermentation, ribose was the highest level of sugar found in mushrooms.

### 3.3. Changes in Total Phenolic Content and Antioxidant Activity in Free and Bound Phenolics during Mushroom Fermentation

The TPC and antioxidant activity of the mushroom fractions are listed in Table 1. The TPC of *L. edodes* free phenolic fraction (LEFPs) and *La. deliciosus* free phenolic fraction (LDFPs), at the beginning of fermentation, were 1.86 ± 0.38 mg GAE/g dm and 1.68 ± 0.14 mg GAE/g dm, respectively. A significant increase in FPs during fermentation occurred within 24 h, with the highest TPC increase of 29% and 27.3% observed for LEFPs and LDFPs, respectively, compared to the unfermented samples (2.40 ± 0.06 mg GAE/g dm and 2.14 ± 0.39 mg GAE/g dm, respectively). Similarly, De Montijo-Prieto et al. (2023) also showed that 24 h fermented samples had the highest level of TPC while investigating the impact of *Lp. plantarum* fermentation on the TPC of ethanolic avocado leaf extracts during 96 h of fermentation [30]. In the present study, the TPC of LEFPs and LDFPs was steady between 24 h and 48 h of fermentation (*p* > 0.05), whereas from 48 h to 72 h, the TPC of the FPs slightly decreased (*p* < 0.05). The decrease in TPC during fermentation has been associated with the degradation of phenolic compounds by *Lp. plantarum* [31]. Comparatively, the bound fractions exhibited notably higher TPC levels than the free fractions did. After ultrasonic–alkaline hydrolysis, a greater amount of BPs were liberated from the cell wall components than FPs. Yeo et al. [11] also found that phenolic compounds were more present in the bound form rather than the free form in lentils. The highest TPC of *L. edodes* bound phenolic fraction (LEBPs) and *La. deliciosus* bound phenolic fraction (LDBPs) were measured at 0 h fermentation and amounted to 4.48 ± 0.26 mg GAE/g dm and 2.93 ± 0.53 mg GAE/g dm, respectively. Previous studies have indicated that the ratio between bound and free phenolic compounds can be altered depending on the processing technologies used, such as fermentation [32]. In the present study, the TPC of BPs in both mushrooms decreased during fermentation. This finding may be due to the availability of endogenous hydrolysing enzymes throughout fermentation and/or the enzymes produced by fermenting microorganisms. The endogenous enzymes benefit from LAB fermentation, which lowers the pH and creates a more optimal environment for their function. The enzymes can break down bonds and facilitate the mobilisation of phenolic compounds from their bound state to their free form [33,34]. LAB can also transform phenolic metabolites and release phenolic compounds from the food matrices during fermentation. This process helps to offset the degradation of the original parent phenolic compounds during fermentation [13].

In the study of Tu et al. (2021), the free phenolic content of *L. edodes* was reported as 7.16 mg GAE/g dry weight (dw), and the bond phenolic content of *L. edodes* was 7.47 ± 0.22 mg GAE/g dw, which corresponds with our results [35]. Similarly, the TPC of the methanolic extract of *L. edodes* was found to be around 2.7 mg GAE/g dw, which closely corresponded with our FPs results [36]. On the other hand, the high TPC of *L. edodes* fractions was opposite to the results reported by Wang et al. [37], who found the highest TPC of BPs of *L. edodes* as 26.67 mg GAE/100 g dm and FPs content as 20.62 mg/GAE 100 dm g. Our study showed that TPC values were 10-times higher than these values. Several factors can account for the observed differences, such as differences in the environment, mushroom variety, location, extraction conditions, substrates, and maturity [36,38]. In a study conducted by Yao et al. (2023), the TPC of ethanolic extract of *L. edodes* was reported as 127 mg GAE/g dw [39]; whereas, in the study by Pehlivan Karakas et al. (2023), the TPC of methanolic extract of *La. deliciosus* was measured as 193 ± 0.77 mg GAE/g dw [40]. The TPC values obtained in our study were lower than those reported in previous studies. It is clear that there are variations in extraction and measurement methods across different studies.

For instance, TPC in a previous study was measured using extracts obtained using 70% (*v*/*v*) ethanol and 80% (*v*/*v*) methanol. Despite these methodological differences, our study confirmed that *L. edodes* and *La. deliciosus* are rich in phenolic compounds.

Phenolic compounds can be used as reducing agents, free-radical scavengers, and singlet oxygen quenchers, and exhibit antioxidant activity primarily because of their ability to transfer hydrogen atoms or donate electrons to free radicals [30]. As shown in Table 1, although LAB fermentation did not increase ABTS radical-scavenging activity, it significantly increased DPPH radical-scavenging activity in the free fractions of both mushrooms, indicating that LAB fermentation may have the potential to enhance the antioxidant activity of mushrooms. An increase in TPC could lead to an enhancement in the antioxidant activity of fermented samples [26]. During the fermentation of both mushrooms, significant positive strong correlations (*p* < 0.05) were observed between DPPH and TPC for LEFPs and LDFPs (R^2^ = 0.95 and R^2^ = 0.95, respectively), and LEBPs and LDBPs (R^2^ = 0.98 and R^2^ = 0.95, respectively). Thus, the higher DPPH activity observed in fermented mushrooms in this study may be attributed to the elevated bioaccessible free total phenolic content modulated by *Lp. plantarum LMG 17673* during lactic acid fermentation. In this study, *Lp. plantarum LMG 17673* fermentation increased the FPs by releasing BFs from the food matrix, potentially enhancing the bioavailability of mushroom phenolics. In addition, the TPC and DPPH radical-scavenging activity of FPs increased, whereas the content in the BP fractions decreased. Similarly, fermentation with selenium-enriched *Lp. plantarum* markedly improves the total phenolic content and antioxidant properties of *Pleurotus eryngii* [32]. Interestingly, the ABTS activity of fermented mushrooms was not correlated with TPC and DPPH activities. The lack of correlation between ABTS levels and TPC and DPPH analysis is similar to the findings in pegaga, a medicinal herb [41], and galangal extracts [42]. The correlation between antioxidant capacities and antioxidant compounds is mostly complicated, as antioxidant capacity is determined by the number of antioxidants, as well as by environmental conditions, the interaction of antioxidants with one another and the matrix, and their physical location and structure [41]. These factors can also be attributed to the ambiguous correlation between ABTS activity and TPC. In addition, the quantified TPC values may include other components that can exert antioxidant activity. Furthermore, assessing antioxidant activity using varying methods based on different mechanisms may yield different results; ABTS relies on the hydrogen atom transfer mechanism, whereas DPPH functions through an electron transfer mechanism [12,34].

### 3.4. The Non-Targeted Analysis of UPLC-Q-TOF-MS Data from Mushroom Fermentation

**Figure 3:**
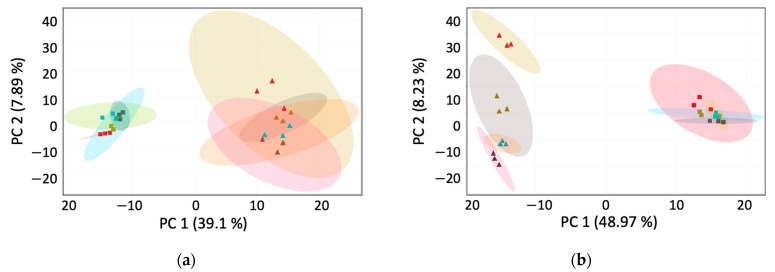
Principal component analysis (PCA) plots with PC 1 and PC 2: (**a**) PCA plot of *L. edodes*; (**b**) PCA plot of *La. deliciosus*. The triangle represents bound phenolics, and the square represents free phenolics. Red colour represents 0 h fermented mushrooms, caramel colour represents 24 h fermented mushrooms, blue colour represents 48 h fermented mushrooms, and brown colour represents 72 h fermented mushrooms.

An unsupervised PCA was applied to gain insights into the metabolic variations among different fermentation time points and phenolic fractions. A non-targeted metabolomics approach using UPLC-Q-TOF-MS datasets was employed for PCA, transforming the dataset into a series of values representing linearly uncorrelated variables. This widely utilised approach in metabolomic studies has allowed us to comprehensively overview metabolite variations among samples. Figure 3a shows the PCA score plot with PC1 and PC2, explaining 46.99% of the total variance (39.1% and 7.89%, respectively), which appeared to be between fermented *L. edodes* fractions. It could be observed that the BPs can be distinctly grouped from FPs. However, overlapping clusters at different fermentation time points in each fraction showed weakly explained variance. Figure 3b shows the PCA score plot with a higher explanation for *La. deliciosus.* PC1 and PC2 explained 57.2% of the total variance (48.97% and 8.23%, respectively), which also appeared to lie between those of fermented *La. deliciosus* fractions. The FPs of unfermented (0 h) *La. deliciosus* exhibited a clear difference from the fermented free fractions. PCA indicated that the form of phenolics was most affected by fermentation, leading to increased differentiation between the fermented and unfermented samples.

**Figure 4:**
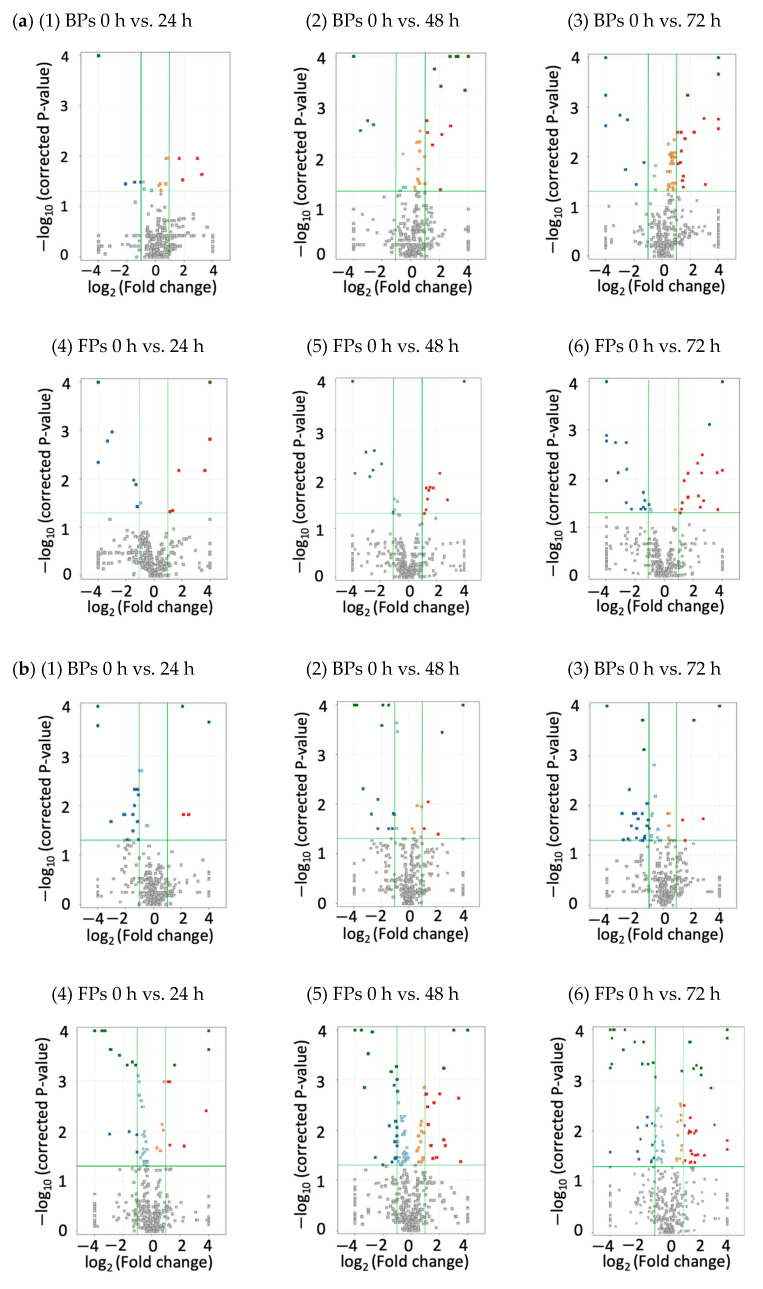
Volcanic plots of differential metabolites in pairwise comparisons between unfermented (0 h) and fermented samples: (**a**) *L. edodes* and (**b**) *La. deliciosus*. BPs; bound phenolics, FPs; free phenolic. The colours in the figure represent the following: grey squares reveal metabolites that show no significant difference between different fermentation time point samples; pale blue or orange squares indicate features that have successfully passed the significance test yet failed to meet the fold-change cut-off criteria. Red squares represent upregulated features, blue squares represent down-regulated features (*p* < 0.05 and FC ≥ 2.0), and green squares in the upper right and left corners represent features (*p* < 0.001 and FC ≥ 2.0).

To further confirm the variance in metabolic profiles among fermented mushrooms, a volcano plot of fermented *L. edodes* (Figure 4a) and *La. deliciosus* (Figure 4b) showed the number of features which changed significantly during the fermentation. The horizontal green line represents the p-test limit, and the features positioned above this line were considered statistically significant (*p* < 0.05). The vertical green lines represent the fold-change limits. The volcano plot shows the passing features (coloured) and non-passing features (grey). Each square, coloured pale blue or orange, corresponds to a feature that has successfully passed the significance test yet failed to meet the fold-change cut-off criteria. During the fermentation of the mushrooms, pairwise group comparisons were conducted by comparing unfermented mushroom samples (0 h fermented) to samples at each further time point, including 24, 48, and 72 h of fermentation. Each red square represents upregulated features, each blue square represents downregulated features (*p* < 0.05 and FC ≥ 2.0), the upper right and left corner green squares represent the features (*p* < 0.001 and FC ≥ 2.0), and grey squares reveal metabolites that have no significant difference between different fermentation time point samples.

**Figure 5:**
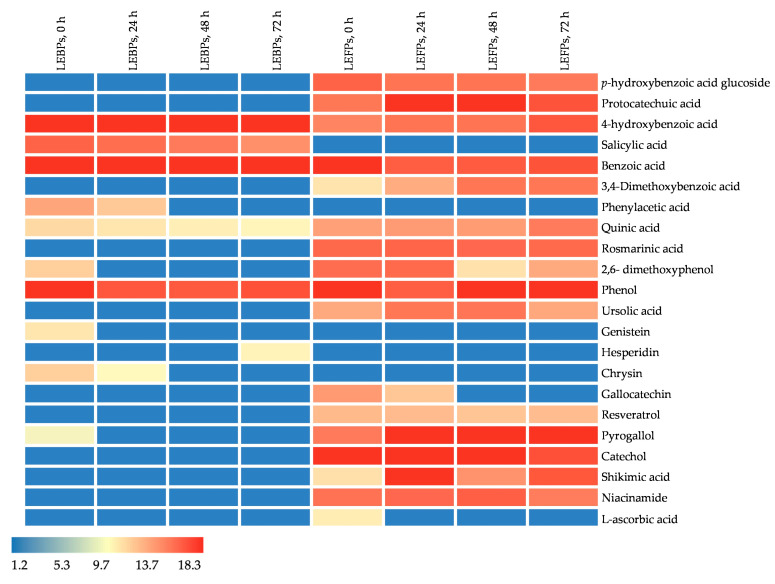
Level of phenolic compounds and vitamins in *L. edodes* fractions during fermentation. LEBPs: *Lentinula edodes* bound phenolics; LEFPs: *Lentinula edodes* free phenolics; LDBPs: *Lactarius delicious* bound phenolics; and LDFPs: *Lactarius delicious* free phenolics. 0 h, 24 h, 48 h, and 72 h represent fermentation time points.

**Figure 6:**
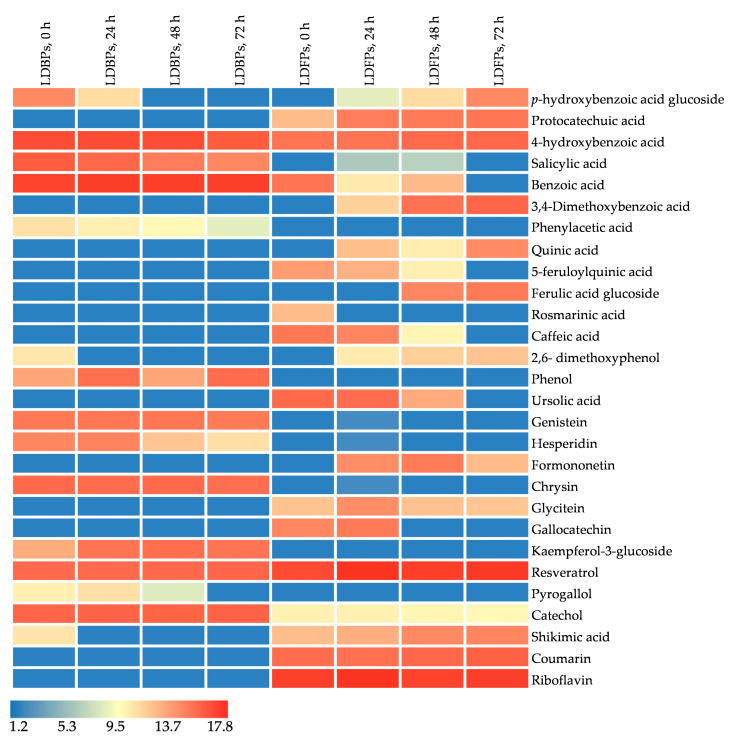
Level of phenolic compounds and vitamins in *La. deliciosus* fractions during fermentation. LEBPs: *Lentinula edodes* bound phenolics; LEFPs: *Lentinula edodes* free phenolics; LDBPs: *Lactarius delicious* bound phenolics; and LDFPs: *Lactarius delicious* free phenolics. 0 h, 24 h, 48 h, and 72 h represent fermentation time points.

A total of 468 features were evaluated in pairwise groups of fermented *L. edodes*. After the volcano plot analysis with *p* < 0.05 and FC ≥ 2.0, 216 out of 468 features were significantly different in *L. edodes* samples during fermentation. The volcano plots of fermented LEBPs showed that the number of features present at 0 h was significantly different from those present at 24, 48, and 72 h, with 17, 45, and 62 metabolites, respectively (as shown in Figure 1, Figure 2 and Figure 3 and Figure 4a) (*p* < 0.05). On the other hand, 0 h fermented LEFPs had 19 significantly different features compared to 24 h fermented LEFPs, which increased to 29 and 44 at 48 h and 72 h, respectively (Figure 4a, Figure 5 and Figure 6) (*p* < 0.05). In addition, 332 of the 469 potential features with significantly different amounts were screened within the pairwise groups for *La. deliciosus* during fermentation (*p* < 0.05 and FC ≥ 2.0). The volcano plot of fermented LDBPs revealed that at 0 h, the number of features was significantly different compared to 24 h, 48 h, and 72 h, with counts of 22, 32, and 49, respectively (Figure 1, Figure 2 and Figure 3 and Figure 4b) (*p* < 0.05). Similarly, LDFPs presenting the number of features at 0 h showed statistically significant differences compared to 24 h, 48 h, and 72 h, with results of 50, 85, and 94, respectively (Figure 4b, Figure 5 and Figure 6) (*p* < 0.05). As the fermentation duration increased, there was a corresponding increase in the number of features in the same fractions that exhibited statistically significant differences in their profiles. These findings suggest that LAB fermentation can alter both the composition and concentration of phenolic compounds, highlighting the fact that bound phenolics are predominantly affected by fermentation. This phenomenon may be linked to lower pH, which has the potential to influence the stability of phenolic compounds, leading to degradation or structural alterations [2]. Li et al. (2019) also observed that the phenolic composition of apple juice could undergo variations during fermentation with *Lp. plantarum* [43]. This alteration may result from the removal or hydrolysis of structural moieties in various phenolic compounds, leading to alterations in phenolic profiles.

### 3.5. Metabolite Profiling and Comparative Analysis of Mushrooms during Different Fermentation Durations via UPLC-Q-TOF-MS/MS

To prevent overestimation of TPC using the spectrophotometric method, we examined variations in individual phenolic compounds that occur during the fermentation process. Secondary metabolites, such as phenolic acids and flavonoids, were detected using Agilent UPLC-ESI-QTOF-MS/MS MassHunter Qualitative Software by considering the MS/MS spectral data. A total of 30 metabolites were tentatively identified in the fermented mushroom fractions (Table 2). Protocatechuic acid, 3,4-dimethoxybenzoic acid, 5-feruloylquinic acid, ferulic acid glucoside, rosmarinic acid, caffeic acid, ursolic acid, formononetin, glycitein, gallocatechin, coumarin, riboflavin, niacinamide, and L-ascorbic acid were only found in the free fractions (LEFPs and LDFPs). With the exception of protocatechuic acid, 3,4-dimethoxybenzoic acid, and phenylacetic acid, all hydroxybenzoic acids were present in the free and bound forms in mushrooms. The cinnamic acid derivatives were found only in their free forms, except for quinic acid. Consistent with our results, *L. edodes* has been reported to contain hydroxybenzoic acid [44], hesperidin [37], 3,4-dimethoxy benzoic acid, glycitein, l-ascorbic acid, riboflavin, niacinamide, salicylic acid, and shikimic acid [45], whereas *La. deliciosus* has been reported to contain hydroxybenzoic acid, pyrogallol, protocatechuic acid [9,46], resveratrol, benzoic acid, kaempferol, chrysin, and ferulic acid [47].

Heat map analysis was used to visualise the samples based on the concentration of bioactive compounds at distinct fermentation time points and phenolic fractions, where the colour scheme from red to blue shows the concentrations in decreasing order. Figure 3 and Figure 4 show significant concentration differences (*p* < 0.05) in *L. edodes* and *La. deliciosus*, identifying 22 and 28 phenolic compounds, respectively, with three vitamins in each. The concentration differences during LAB fermentation can be explained by changes in the content and profile of bioactive compounds, modification of the parent phenolic compounds, and structural breakdown of the mushroom cell walls [48]. In our study, certain compounds that became undetectable or reduced within FPs during fermentation could be associated with the metabolic activities of *Lp. plantarum LMG 17673*. For instance, there was a decrease in p-hydroxybenzoic acid glucoside in both LEFPs and LDFPs, which corresponded to a higher accumulation of its reduced metabolite, 4-hydroxybenzoic acid. As a result of this concentration difference, the concentration of quinic acid increased in both LEFPs and LDFPs. In contrast, that of 5-feruloylquinic acid, a derivative of quinic acid, decreased during LAB fermentation. This decrease might have contributed to the increase in quinic acid content, suggesting a potential conversion. Similarly, the concentration of quinic acid was significantly higher in avocado seeds fermented with *Lp. plantarum,* indicating possible hydrolysis of hydroxycinnamic acids [49]. Additionally, hydroxycinnamic acids, such as caffeic acid, can undergo transformation into different compounds facilitated by reductase enzymes. This process may lead to the metabolism of dihydrocaffeic acids, which can further undergo decarboxylation to produce vinyl derivatives such as vinyl phenol and vinyl guaiacol [30,50]. Previous studies have reported that the *Lp. plantarum* may contribute to the degradation of phenolic compounds through processes such as depolymerisation, hydrolysis, decarboxylase-mediated metabolism, and reductase activity [51].

Fermentation increased the levels of certain components, such as protocatechuic acid, 4-hydroxybenzoic acid, 3,4-dimethoxybenzoic acid, quinic acid, phenol, pyrogallol, and shikimic acid in *L. edodes* FPs. Likewise, in the free fraction of *La. deliciosus*, the concentrations of protocatechuic acid, 4-hydroxybenzoic acid, salicylic acid, 3,4-dimethoxybenzoic acid, quinic acid, ferulic acid glucoside, 2,6-dimethoxyphenol, formononetin, glycitein, resveratrol, shikimic acid, and coumarin were significantly higher than those in the initial stages of fermentation. Furthermore, the biotransformation of ferulic acid to vanillin has been well-studied in various bacterial species, including LAB [52]. However, we found increased ferulic acid glucoside levels in LDFPs without identifying vanillin, possibly because of its low concentration below the threshold (score < 75). Moreover, during *L. edodes* fermentation, quinic acid, 2,6-dimethoxyphenol, phenol, and pyrogallol concentrations decreased in the bound fraction but increased in the free fraction. Similarly, during *La. deliciosus* fermentation, the levels of 4-hydroxybenzoic acid, salicylic acid, 2,6-dimethoxyphenol, and shikimic acid decreased in the bound fraction and increased in the free fraction. This phenomenon can be explained by the ability of LAB to break down large molecules in favour of phenolic compounds in mushrooms, which may include breaking down cell wall polysaccharides and proteins, wherein bound phenolics are linked [53]. Thus, LAB fermentation can potentially enhance biological activity by converting bound phenolics to a free state. Additionally, LAB fermentation can biotransform complex phenolic compounds and flavonoids into simple bioactive compounds, such as phenolic acids, thereby increasing their bioavailability [54].

## 4. Conclusions

This study demonstrated the efficiency of LAB fermentation in the biotransformation of phenolic compounds. *Lp. plantarum LMG 17673* was successfully fermented for 72 h on the substrate of *L. edodes* and *La. deliciosus*, resulting in a high viable cell count. *L. edodes* and *La. deliciosus* were rich in bound and free phenolics, with the former fraction being notably more abundant. LAB fermentation enhanced the releasing of bound phenolics and modified their parent phenolic compounds and structure. The phenolic compound composition and concentration changes were assessed using UPLC-MS-TOF-MS/MS. This study detected thirty phenolic compounds in the mushroom fractions during LAB fermentation. Notably, DPPH scanning activity analysis indicated that the release of bound phenolics significantly contributed to antioxidant activity through fermentation. Hence, this study demonstrated the effect of bound phenolics on the bioaccessibility of mushrooms. Subsequent investigations concerning the conversion mechanism of phenolic compounds within mushrooms through LAB fermentation should be pursued in future studies. 

## Figures and Tables

**Table 1:** Effect of fermentation on TPC and antioxidant activity of free and bound phenolic fractions of fermented mushrooms.

Fermentation Time	TPC (mg GAE/g)				DPPH (mg TE/g)				ABTS (mg TE/g)			
	*L. edodes*		*La. deliciosus*		*L. edodes*		*La. deliciosus*		*L. edodes*		*La. deliciosus*	
	Free	Bound	Free	Bound	Free	Bound	Free	Bound	Free	Bound	Free	Bound
0 h	1.86 ± 0.04 ^c^	4.48 ± 0.08 ^a^	1.68 ± 0.01 ^c^	2.93 ± 0.05 ^a^	2.55 ± 0.11 ^c^	6.54 ± 0.26 ^a^	2.26 ± 0.13 ^b^	1.81 ± 0.14 ^a^	6.29 ± 0.21 ^b^	23.68 ± 1.51 ^a^	5.69 ± 0.14 ^a^	26.71 ± 1.43 ^b^
24 h	2.40 ± 0.01 ^a^	4.10 ± 0.06 ^b^	2.14 ± 0.04 ^a^	2.36 ± 0.04 ^b^	3.77 ± 0.10 ^a^	5.55 ± 0.16 ^b^	2.88 ± 0.36 ^a^	1.66 ± 0.15 ^ab^	5.90 ± 0.45 ^b^	24.04 ± 1.61 ^a^	5.56 ± 0.31 ^a^	28.05 ± 0.1 ^a^
48 h	2.33 ± 0.04 ^a^	3.79 ± 0.02 ^c^	2.09 ± 0.07 ^a^	2.35 ± 0.02 ^b^	3.36 ± 0.13 ^b^	5.21 ± 0.11 ^b^	2.64 ± 0.03 ^ab^	1.44 ± 0.12 ^bc^	5.61 ± 0.71 ^b^	23.40 ± 1.5 ^a^	5.67 ± 1.23 ^a^	25.45 ± 0.23 ^c^
72 h	2.14 ± 0.04 ^b^	3.50 ± 0.04 ^d^	1.88 ± 0.03 ^b^	2.21 ± 0.04 ^b^	3.30 ± 0.14 ^b^	4.24 ± 0.09 ^c^	2.54 ± 0.06 ^ab^	1.31 ± 0.11 ^c^	6.10 ± 0.29 ^a^	22.34 ± 2.21 ^a^	5.47 ± 0.43 ^a^	25.5 ± 0.14 ^c^

TPC: total phenolic content; GAE: gallic acid equivalent antioxidant capacity; DPPH: 2,2-diphenyl-1-picrylhydrazyl; TE: trolox equivalent; ABTS: 2,2′azino-bis(3-ethylbenzthiazoline-6-sulphonic acid) diammonium salt. Different lowercase letters in the same column indicate significant differences between the samples in the same column (*p* < 0.05). Values are presented as mean ± SD (*n* = 3).

**Table 2:** Tentative identification of phenolic compounds, terpenic acids, and vitamins in the fermented *L. edodes* and *La. deliciosus* fractions.

No	Proposed Compound	Molecular Formula	RT (min)	Adduct	Molecular Weight	Theoretical (*m*/*z*)	Observed (*m*/*z*)	Mass Error (ppm)	MS/MS Product Ions Collision Energies			Mushroom Species	
									10 V	20 V	40 V	*L. edodes*	*La. deliciosus*
	Phenolic acids												
	i. Hydroxybenzoic acids												
1	*p*-hydroxybenzoic acid glucoside	C_13_H_16_O_8_	13.42	(M−H)−	300.085	299.0779	299.0772	2.1	299	-	106	All-LEBPs All-LEFPs	LDBPs-0 h LDBPs-24 h All-LDFPs
2	Protocatechuic acid	C_7_H_6_O_4_	4.30	(M−H)−	154.0264	153.0191	153.0193	−1.47	-	111	-	All-LEFPs	All-LDFPs
3	4-hydroxybenzoic acid *	C_7_H_6_O_3_	7.91	(M−H)−	138.0318	137.0245	137.0244	0.76	109	111, 137	-	All-LEBPs All-LEFPs	All-LDBPs All-LDFPs
4	Salicylic acid *	C_7_H_6_O_3_	19.83	(M−H)−	138.0317	137.0238	137.0244	−4.6	-	111	-	All-LEBPs	All-LDBPs LDFPs-24 h LDFPs-48 h
5	Benzoic acid	C_7_H_6_O_2_	9.6	(M−H)−	122.0368	121.0295	121.0295	0.35	120	121	-	All-LEBPs All-LEFPs	All-LDBPs LDFPs-0 h LDFPs-24 h LDFPs-48 h
6	3,4-Dimethoxybenzoic acid	C_9_H_10_O_4_	6.99	(M−H)−	182.0579	181.0506	181.0505	−0.16	101, 141	120	-	All-LEFPs	LDFPs-24 h LDFPs-48 h LDFPs-72 h
7	Phenylacetic acid *	C_8_H_8_O_2_	11.41	(M−H)−	136.0516	135.0452	135.0452	0.14	135	117, 109	-	LEBPs-0 h LEBPs-24 h	All-LDBPs
	ii. Hydroxycinnamic acids												
8	Quinic acid	C_7_H_12_O_6_	7.25	(M+HCOO)−	192.0638	237.057	237.062	1.95	156, 126	-	-	All-LEBPs All-LEFPs	LDFPs-24 h LDFPs-48 h LDFPs-72 h
9	5-feruloylquinic acid	C_17_H_20_O_9_	4.35	(M+HCOO)−	368.1093	413.1227	413.0143	-3.8	312	-	-	-	LDFPs-0 h LDFPs-24 h LDFPs-48 h
11	Rosmarinic acid	C_18_H_16_O_8_	11.18	(M+HCOO)−	360.087	405.0809	405.0827	−4.5	211	191, 227	139, 171	All-LEFPs	LDFPs-0 h
12	Caffeic acid	C_9_H_8_O_4_	10.20	(M−H)−	180.0425	179.0352	179.0332	3.3	124	165	-	-	LDFPs-0 h LDFPs-24 h LDFPs-48 h
	Phenols												
13	2,6-dimethoxyphenol	C_8_H_10_O_3_	8.77	(M+HCOO)−	154.0628	199.06	199.061	0.32	123	128	-	LEBPs-0 h All-LEFPs	LDBPs-0 h LDFPs-24 h LDFPs-48 h LDFPs-72 h
14	Phenol	C_6_H_6_O	30.87	(M+HCOO)−	94.0416	139.039	139.0398	−2.61	86	-	-	All-LEBPs All-LEFPs	All-LDBPs
	Terpenic acids												
15	Ursolic acid *	C_30_H_48_O_3_	35.81	(M−H)− (M+HCOO)−	456.3603	455.3544 501.3584	455.3531 501.3585	2.82 −0.33	391 297, 337	255 152	163, 283 435	All-LEBPs	LDFPs-0 h LDFPs-24 h LDFPs-48 h
	Flavonoids												
16	Genistein	C_15_H_10_O_5_	28.13	(M−H)−	270.053	269.0457	269.0455	0.42	151, 107	119	187	LEBPs-0 h	All-LDBPs
18	Formononetin	C_16_H_12_O_4_	29.35	(M−H)−	268.0733	267.066	267.0663	−1	251	-	-	-	LDFPs-24 h LDFPs-48 h LDFPs-72 h
20	Glycitein	C_16_H_12_O_5_	30.91	(M+HCOO)−	284.0745	329.0778	329.0774	−0.92	285	-	-	-	All-LDFPs
21	Gallocatechin	C_15_H_14_O_7_	4.22	(M−H)−	306.0718	305.0702	305.0645	−2.8	156	-	-	LEFPs-0 h LEFPs-24 h	LDFPs-0 h LDFPs-24 h
22	Kaempferol-3-glucoside	C_21_H_20_O_11_	22.99	(M−H)−	448.1001	447.0928	447.0933	−1.07	224, 115	-	-	-	All-LDBPs
	Stilbenes												
23	Resveratrol	C_14_H_12_O_3_	29.02	(M−H)−	228.0785	227.0713	227.0714	−0.49	183, 199	155, 141	139, 167	All-LEFPs	All-LDBPs All-LDFPs
	Other polyphenols												
24	Pyrogallol *	C_6_H_6_O_3_	3.57	(M−H)− (M+HCOO)−	126.0318	125.0244 171.03	125.0244 171.0299	0.21 0.59	124 103	108, 124 -	- -	LEBPs-0 h All-LEFPs	LDBPs-0 h LDBPs-24 h LDBPs-48 h
25	Catechol	C_6_H_6_O_2_	5.17	(M−H)−	110.0366	109.032	109.0294	0.3	101	-	-	All-LEFPs	All-LDBPs All-LDFPs
26	Shikimic acid	C_7_H_10_O_5_	2.9	(M−H)− (M+HCOO)−	174.0529	173.0457 219.0509	173.0455 219.051	0.82 −0.37	157, 109 113	- -	- -	All-LEFPs	LDBPs-0 h All-LDFPs
27	Coumarin	C_9_H_6_O_2_	15.5	(M−H)−	146.0368	145.0295	145.0245	0.12	142	108	-	-	All-LDFPs
	Vitamins												
28	Riboflavin	C_17_H_20_N_4_O_6_	16.22	(M−H)−	376.1384	375.131	375,131	−0.08	255, 129	212, 165	198, 184	-	All-LDFPs
29	Niacinamide	C_6_H_6_N_2_O	15.62	(M+HCOO)−	122.048	167.0462	167.0462	−0.23	124, 128	108	-	All-LEFPs	-
30	L-ascorbic acid	C_6_H_8_O_6_	1.66	(M−H)−	176.032	175.0247	175.0248	−0.92	117, 175	-	-	LEFPs-0 h	-

MS/MS: Tandem mass spectrometry; LEBPs: *Lentinula edodes* bound phenolics; LEFPs: *Lentinula edodes* free phenolics; LDBPs: *Lactarius delicious* bound phenolics; LDFPs: *Lactarius delicious* free phenolics. “All” represents all fermentation time points in the mentioned fraction. 0 h, 24 h, 48 h, and 72 h represent fermentation time points. “-” is not detected. “RT” stands for retention time. * The compounds were verified using authentic standards.

## Data Availability

The original contributions presented in the study are included in the article and Supplementary Materials, further inquiries can be directed to the corresponding author.

## Supplementary Materials

The following supporting information can be downloaded at: https://www.mdpi.com/article/10.3390/foods13111616/s1, Table S1: Changes in free sugars and organic acids in fermented mushrooms during fermentation.
