# Supplementary material for: Impact of Lactic Acid Bacteria Fermentation Based on Biotransformation of Phenolic Compounds and Antioxidant Capacity of Mushrooms
Source: Foods. 2024 May 23;13(11):1616. doi: 10.3390/foods13111616 (PMC11172137; doi:10.3390/foods13111616)
Supplement: Supplementary file 1 [file foods-13-01616-s001.zip › foods-2984367-supplementary.pdf]

Table S1. Changes in free sugars and organic acids in fermented mushrooms during fermentation

**Free sugars and organic acids (mg/g dm)**	***L. edodes***				***La. deliciosus***				LOD (mg/L)	LOQ (mg/L)
	**Fermentation time (hours)**									
	**0**	**24**	**48**	**72**	**0**	**24**	**48**	**72**		
D(+)glucose	32.94±0.85	D	ND	ND	41.55±0.32^{a}	33.47±0.83^{a}	ND	ND	26.30	79.72
D(-)mannitol	221.46±6.16^{a}	23.90±0.15^{b}	2.93±0.41^{c}	ND	181.80±0.18^{a}	31.90±2.24^{b}	8.24±0.70^{c}	0.48±0.08^{d}	43.79	132.70
D(-)ribose	51.14±0.76^{a}	35.14±0.13^{b}	48.68±6.63^{a}	37.24±0.49^{b}	60.67±4.26^{b}	68.47±8.63^{ab}	76.40±2.26^{a}	78.48±5.26^{a}	29.57	74.72
Trehalose	10.29±0.64^{a}	0.86±0.15^{b}	ND	ND	25.43±0.11^{a}	0.79±0.03^{b}	0.89±0.02^{b}	0.82±0.02^{b}	29.58	89.63
Malic acid	54.72±2.28^{a}	2.82±0.86^{b}	1.64±0.26^{b}	ND	66.12±3.15	ND	ND	ND	6.19	20.64
Succinic acid	177.69±7.44^{a}	9.42±0.13^{b}	11.88±3.09^{b}	8.64±0.12^{b}	300.59±3.65^{a}	20.88±1.16^{b}	9.15±1.44^{b}	6.11±0.57^{b}	4.02	13.39
Fumaric acid	1.11±0.47	ND	ND	ND	1.20±0.01	D	D	ND	2.00	6.66
Citric acid	10.91±2.17^{a}	4.35±0.83^{b}	5.27±2.54^{b}	4.59±0.65^{b}	25.22±1.87^{a}	3.59±0.08^{c}	6.71±0.66^{b}	8.82±0.18^{b}	6.48	21.61
Lactic acid	106.33±3.88^{c}	525.76±8.1^{b}	574.00±19.03^{b}	697.60±9.34^{a}	136.07±5.44^{b}	1576.79±19.30^{a}	1480.47±15.23^{a}	1446.03±21.20^{a}	6.88	22.92
Formic acid	10.47±0.44^{c}	13.64±0.61^{b}	16.13±4.23^{a}	12.34±0.56^{b}	12.89±1.20^{b}	20.15±2.08^{a}	19.72±2.33^{a}	19.71±0.51^{a}	1.84	6.12

LOD: Limit of detection; LOQ: Limit of quantitation
